# 3-(3-Chloro­phen­yl)-1-methyl-3,3a,4,9b-tetra­hydro-1*H*-chromeno[4,3-*c*]isoxazole-3a-carbonitrile

**DOI:** 10.1107/S1600536811007495

**Published:** 2011-03-05

**Authors:** K. Swaminathan, K. Sethusankar, G. Murugan, M. Bakthadoss

**Affiliations:** a Department of Physics, RKM Vivekananda College (Autonomous), Chennai 600 004, India; b Department of Organic Chemistry, University of Madras, Maraimalai Campus, Chennai 600 025, India

## Abstract

In the title compound, C_18_H_15_ClN_2_O_2_, the five-membered isoxazole ring adopts an envelope conformation [the deviation of the N atom is 0.3154 (15) Å] and the six-membered pyran ring adopts a half-chair conformation. The mean plane through all atoms of the isoxazole ring forms dihedral angles of 47.98 (8)° with the mean plane of the chromene ring system and 75.10 (9)° with the chloro­benzene ring.

## Related literature

For the synthesis of tricyclic chromenoisoxazolidines, see: Bakthadoss & Murugan (2010 ▶). For uses of isoxazole derivatives, see: Loh *et al.* (2010 ▶); Winn *et al.* (1976 ▶). For a related structure, see: Gunasekaran *et al.* (2010 ▶). For puckering parameters, see: Cremer & Pople (1975 ▶).
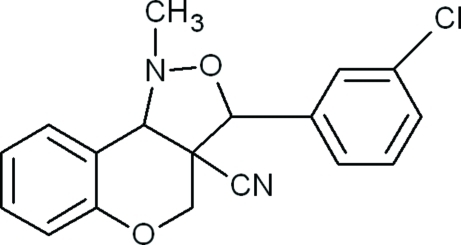


## Experimental

### 

#### Crystal data

C_18_H_15_ClN_2_O_2_
*M* *_r_* = 326.77
Monoclinic, 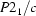

*a* = 10.0141 (4) Å
*b* = 9.2358 (3) Å
*c* = 17.5945 (6) Å
β = 102.354 (2)°
*V* = 1589.60 (10) Å^3^
*Z* = 4
Mo *K*α radiation
μ = 0.25 mm^−1^
*T* = 295 K
0.30 × 0.25 × 0.20 mm

#### Data collection

Bruker Kappa APEXII CCD diffractometer
Absorption correction: multi-scan (*SADABS*; Sheldrick, 1996 ▶) *T*_min_ = 0.928, *T*_max_ = 0.952
20534 measured reflections
4926 independent reflections
3390 reflections with *I* > 2σ(*I*)
*R*_int_ = 0.026

#### Refinement

*R*[*F* ^2^ > 2σ(*F* ^2^)] = 0.051
*wR*(*F* ^2^) = 0.151
*S* = 1.04
4926 reflections
209 parameters
H-atom parameters constrained
Δρ_max_ = 0.61 e Å^−3^
Δρ_min_ = −0.63 e Å^−3^

### 

Data collection: *APEX2* (Bruker, 2004 ▶); cell refinement: *SAINT* (Bruker, 2004 ▶); data reduction: *SAINT*; program(s) used to solve structure: *SHELXS97* (Sheldrick, 2008 ▶); program(s) used to refine structure: *SHELXL97* (Sheldrick, 2008 ▶); molecular graphics: *ORTEP-3* (Farrugia, 1997 ▶); software used to prepare material for publication: *SHELXL97* and *PLATON* (Spek, 2009 ▶).

## Supplementary Material

Crystal structure: contains datablocks global, I. DOI: 10.1107/S1600536811007495/rk2265sup1.cif

Structure factors: contains datablocks I. DOI: 10.1107/S1600536811007495/rk2265Isup2.hkl

Additional supplementary materials:  crystallographic information; 3D view; checkCIF report

## Figures and Tables

**Fig. 1.**
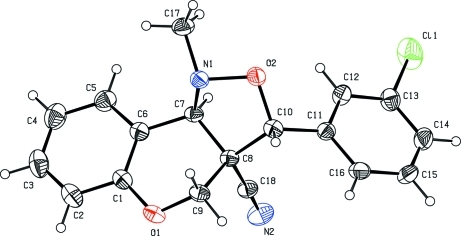
The molecular structure of the title compound with the atom numbering scheme. Displacement ellipsoids are drawn at 30% probability level. H atoms are presented as a small spheres of arbitary radius.

## supplementary crystallographic information

### Comment

Using Baylis-Hillman derivatives through *in situ* formation of nitrones followed by an intramolecular [3+2] dipolar cycloaddition reaction sequence is a novel and simple method of synthesizing tricyclic chromenoisoxozolidine frameworks. The new [3+2] cycloaddition reaction leads to a novel class of angularly substituted fused tricyclic chromenoisoxazolidines, creating two rings and three contiguous stereocenters, one of them being a tetrasubstituted carbon center. (Bakthadoss & Murugan, 2010). Benzopyran and isoxazolidine derivatives are well known for their biological activity and proven medicinal utility. For example, benzopyran derivatives possess antipsychotic and antidepressant activities (Winn *et al.*, 1976). Isoxazolidine and isoxazole sulfonamide are found to inhibit *HIV*-1 infection in human *CD*4+ lymphocytic *T* cells (Loh *et al.*, 2010).

The title compound C_18_H_15_N_2_O_2_Cl comprises a chromenoisoxazole ring system attached to a chlorobenzene ring and a carbonitrile group. The X-ray analysis confirms the molecular structure and atom connectivity as illustrated at (Fig. 1). In the isoxazole ring (N1/O2/C7/C8/C10), the deviation of atom N1 is -0.3154 (15)Å. This ring adopts an *envelope* conformation with puckering parameters (Cremer & Pople, 1975) q_2_ = 0.5041 (15)Å and φ_2_ = 44.23 (17)°. The dihedral angle between the chromeno ring system (O1/C1–C9) and the isoxazole ring(N1/O2/C7/C8/C10) is 47.98 (8)°. The isoxazole ring (N1/O2/C7/C8/C10) also forms a dihedral angle of 75.10 (9)° with the the chlorobenzene ring (C11–C16).

In the chromeno ring system, the dihedral angle between the pyran ring (O1/C1/C6-C9) and the benzene ring(C1-C6) is 3.50 (9)°. The deviation of atom C9 from the mean plane of the pyran ring is 0.3068 (17)Å. The pyran ring adopts *half chair* conformation (H-form), with puckering parameters (Cremer & Pople, 1975) q_2_ = 0.3501 (17)Å, q_3_ = -0.3037 (16)Å and φ_2_ = 94.7 (2)°. The pyran ring (O1/C1/C6–C9) also forms an interplanar angle of 49.31 (8)° with the isoxazole ring (N1/O2/C7/C8/C10). The chlorobenzene ring(C11–C16) forms an interplanar angle of 51.98 (8)° with the mean plane of the fused isoxazole–pyran ring system (N1/O1/O2/C1/C6–C10). Also, the dihedral angle between the chlorobenzene ring (C11–C16) and the chromeno ring system (O1/C1–C9) is 39.95 (7)°. The title compound exhibits structural similarities with other reported related structures (Gunasekaran *et al.*, 2010). There are no classic hydrogen bonds.

### Experimental

A mixture of the compound (*E*)-2-((2-formylphenoxy) methyl)-3-(3-chlorophenyl) acrylonitrile (1.0 mmol) with *N*-methylhydroxylamine hydrochloride (1.1 mmol), pyridine (0.24 ml, 3 mmol) and ethanol (5 ml) were placed in a round bottom flask and refluxed for 6 h. After completion of the reaction as indicated by *TLC*, the reaction mixture was concentrated under reduced pressure. The crude product was diluted with water (10 ml) and dilute HCl (5 ml) and extracted with ethylacetate (20 ml). The organic layer was washed with brine solution (10 ml) and concentrated. The crude product was purified by column chromatography to provide the pure desired compound, as a colourless solid.

### Refinement

All hydrogen atoms were placed in calculated positions with C—H = 0.93–0.98Å and refined in riding model with fixed isotropic displacement parameters: *U*_iso_(H) = 1.5*U*_eq_(C) for methyl group and *U*_iso_(H) = 1.2*U*_eq_(C) for other groups.

### Crystal data

**Table:**

C_18_H_15_ClN_2_O_2_	*F*(000) = 680
*M**_r_* = 326.77	*D*_x_ = 1.365 Mg m^−^^3^
Monoclinic, *P*2_1_/*c*	Mo *K*α radiation, λ = 0.71073 Å
Hall symbol: -P 2ybc	Cell parameters from 4926 reflections
*a* = 10.0141 (4) Å	θ = 1.0–25.0°
*b* = 9.2358 (3) Å	µ = 0.25 mm^−^^1^
*c* = 17.5945 (6) Å	*T* = 295 K
β = 102.354 (2)°	Block, colourless
*V* = 1589.60 (10) Å^3^	0.30 × 0.25 × 0.20 mm
*Z* = 4	

### Data collection

**Table:**

Bruker Kappa APEXII CCD diffractometer	4926 independent reflections
Radiation source: fine-focus sealed tube	3390 reflections with *I* > 2σ(*I*)
graphite	*R*_int_ = 0.026
ω scans	θ_max_ = 30.7°, θ_min_ = 2.1°
Absorption correction: multi-scan (*SADABS*; Sheldrick, 1996)	*h* = −14→13
*T*_min_ = 0.928, *T*_max_ = 0.952	*k* = −13→12
20534 measured reflections	*l* = −25→25

### Refinement

**Table:**

Refinement on *F*^2^	Primary atom site location: structure-invariant direct methods
Least-squares matrix: full	Secondary atom site location: difference Fourier map
*R*[*F*^2^ > 2σ(*F*^2^)] = 0.051	Hydrogen site location: inferred from neighbouring sites
*wR*(*F*^2^) = 0.151	H-atom parameters constrained
*S* = 1.04	*w* = 1/[σ^2^(*F*_o_^2^) + (0.0641*P*)^2^ + 0.525*P*] where *P* = (*F*_o_^2^ + 2*F*_c_^2^)/3
4926 reflections	(Δ/σ)_max_ < 0.001
209 parameters	Δρ_max_ = 0.61 e Å^−^^3^
0 restraints	Δρ_min_ = −0.63 e Å^−^^3^

### Special details

**Table:**

Geometry. All s.u.'s (except the s.u. in the dihedral angle between two l.s. planes) are estimated using the full covariance matrix. The cell s.u.'s are taken into account individually in the estimation of s.u.'s in distances, angles and torsion angles; correlations between s.u.'s in cell parameters are only used when they are defined by crystal symmetry. An approximate (isotropic) treatment of cell s.u.'s is used for estimating s.u.'s involving l.s. planes.
Refinement. Refinement of *F*^2^ against ALL reflections. The weighted *R*-factor *wR* and goodness of fit *S* are based on *F*^2^, conventional *R*-factors *R* are based on *F*, with *F* set to zero for negative *F*^2^. The threshold expression of *F*^2^ > σ(*F*^2^) is used only for calculating *R*-factors(gt) *etc*. and is not relevant to the choice of reflections for refinement. *R*-factors based on *F*^2^ are statistically about twice as large as those based on *F*, and *R*-factors based on ALL data will be even larger.

### Fractional atomic coordinates and isotropic or equivalent isotropic displacement parameters (Å^2^)

**Table:**

	*x*	*y*	*z*	*U*_iso_*/*U*_eq_	
C1	0.71130 (16)	0.55309 (17)	0.60426 (9)	0.0414 (3)	
C2	0.7392 (2)	0.5485 (2)	0.53022 (11)	0.0558 (5)	
H2	0.6764	0.5081	0.4890	0.067*	
C3	0.8604 (2)	0.6042 (3)	0.51838 (13)	0.0661 (6)	
H3	0.8786	0.6031	0.4687	0.079*	
C4	0.9552 (2)	0.6618 (3)	0.57932 (14)	0.0666 (6)	
H4	1.0375	0.6980	0.5710	0.080*	
C5	0.92754 (19)	0.6654 (2)	0.65242 (12)	0.0527 (4)	
H5	0.9924	0.7029	0.6936	0.063*	
C6	0.80402 (15)	0.61383 (17)	0.66604 (9)	0.0391 (3)	
C7	0.77527 (14)	0.61740 (15)	0.74613 (8)	0.0347 (3)	
H7	0.8576	0.5912	0.7847	0.042*	
C8	0.65603 (14)	0.51912 (15)	0.75457 (8)	0.0345 (3)	
C9	0.54349 (15)	0.53043 (18)	0.68020 (9)	0.0400 (3)	
H9A	0.4677	0.4683	0.6851	0.048*	
H9B	0.5100	0.6292	0.6743	0.048*	
C10	0.60629 (16)	0.58987 (17)	0.82461 (9)	0.0392 (3)	
H10	0.5095	0.6157	0.8079	0.047*	
C11	0.62489 (15)	0.49933 (17)	0.89708 (9)	0.0382 (3)	
C12	0.74430 (16)	0.50552 (19)	0.95373 (10)	0.0435 (4)	
H12	0.8145	0.5681	0.9483	0.052*	
C13	0.75772 (19)	0.4174 (2)	1.01837 (10)	0.0503 (4)	
C14	0.6564 (2)	0.3247 (2)	1.02857 (12)	0.0584 (5)	
H14	0.6676	0.2664	1.0726	0.070*	
C15	0.5385 (2)	0.3195 (2)	0.97269 (12)	0.0621 (5)	
H15	0.4687	0.2569	0.9788	0.075*	
C16	0.52159 (19)	0.4062 (2)	0.90713 (11)	0.0512 (4)	
H16	0.4406	0.4020	0.8697	0.061*	
C17	0.8261 (2)	0.86941 (19)	0.78734 (11)	0.0531 (4)	
H17A	0.7878	0.9520	0.8080	0.080*	
H17B	0.8582	0.8975	0.7418	0.080*	
H17C	0.9010	0.8321	0.8257	0.080*	
C18	0.69897 (16)	0.36725 (17)	0.76710 (10)	0.0405 (3)	
N1	0.72137 (14)	0.75768 (14)	0.76661 (7)	0.0396 (3)	
N2	0.72938 (17)	0.24949 (17)	0.77403 (11)	0.0618 (4)	
O1	0.59142 (12)	0.48990 (13)	0.61273 (7)	0.0470 (3)	
O2	0.68515 (14)	0.71908 (12)	0.84078 (7)	0.0481 (3)	
Cl1	0.90798 (6)	0.42666 (9)	1.08998 (4)	0.0900 (2)	

### Atomic displacement parameters (Å^2^)

**Table:**

	*U*^11^	*U*^22^	*U*^33^	*U*^12^	*U*^13^	*U*^23^
C1	0.0467 (8)	0.0382 (8)	0.0375 (8)	0.0095 (6)	0.0049 (6)	−0.0010 (6)
C2	0.0680 (12)	0.0572 (11)	0.0404 (9)	0.0170 (9)	0.0079 (8)	−0.0024 (8)
C3	0.0852 (14)	0.0674 (13)	0.0533 (11)	0.0204 (11)	0.0318 (11)	0.0081 (10)
C4	0.0685 (12)	0.0649 (13)	0.0749 (14)	0.0032 (10)	0.0344 (11)	0.0058 (11)
C5	0.0475 (9)	0.0522 (10)	0.0595 (11)	−0.0014 (8)	0.0142 (8)	−0.0013 (8)
C6	0.0402 (7)	0.0350 (7)	0.0406 (8)	0.0046 (6)	0.0055 (6)	0.0011 (6)
C7	0.0346 (6)	0.0300 (7)	0.0355 (7)	0.0006 (5)	−0.0015 (5)	−0.0001 (5)
C8	0.0353 (7)	0.0285 (6)	0.0369 (7)	0.0029 (5)	0.0016 (5)	−0.0002 (5)
C9	0.0341 (7)	0.0415 (8)	0.0406 (8)	0.0013 (6)	−0.0007 (6)	−0.0047 (6)
C10	0.0401 (7)	0.0358 (7)	0.0402 (8)	0.0050 (6)	0.0053 (6)	−0.0013 (6)
C11	0.0402 (7)	0.0351 (7)	0.0404 (8)	0.0004 (6)	0.0111 (6)	−0.0026 (6)
C12	0.0395 (7)	0.0456 (9)	0.0454 (8)	−0.0006 (7)	0.0091 (6)	0.0074 (7)
C13	0.0550 (10)	0.0499 (10)	0.0453 (9)	0.0078 (8)	0.0091 (7)	0.0076 (7)
C14	0.0899 (14)	0.0380 (9)	0.0523 (10)	−0.0021 (9)	0.0262 (10)	0.0051 (8)
C15	0.0824 (14)	0.0455 (10)	0.0662 (12)	−0.0269 (10)	0.0331 (11)	−0.0089 (9)
C16	0.0518 (9)	0.0503 (10)	0.0517 (10)	−0.0143 (8)	0.0119 (8)	−0.0137 (8)
C17	0.0676 (11)	0.0342 (8)	0.0540 (10)	−0.0085 (8)	0.0052 (8)	−0.0031 (7)
C18	0.0404 (7)	0.0326 (7)	0.0471 (8)	0.0010 (6)	0.0061 (6)	0.0004 (6)
N1	0.0513 (7)	0.0293 (6)	0.0360 (6)	0.0008 (5)	0.0045 (5)	0.0011 (5)
N2	0.0636 (10)	0.0364 (8)	0.0845 (12)	0.0068 (7)	0.0135 (9)	0.0031 (8)
O1	0.0452 (6)	0.0517 (7)	0.0392 (6)	−0.0014 (5)	−0.0016 (5)	−0.0116 (5)
O2	0.0751 (8)	0.0314 (6)	0.0395 (6)	−0.0028 (5)	0.0158 (5)	−0.0031 (4)
Cl1	0.0704 (4)	0.1252 (6)	0.0635 (4)	0.0115 (4)	−0.0098 (3)	0.0284 (3)

### Geometric parameters (Å, °)

**Table:**

C1—O1	1.371 (2)	C10—O2	1.4257 (19)
C1—C6	1.388 (2)	C10—C11	1.503 (2)
C1—C2	1.390 (2)	C10—H10	0.9800
C2—C3	1.375 (3)	C11—C12	1.384 (2)
C2—H2	0.9300	C11—C16	1.385 (2)
C3—C4	1.378 (3)	C12—C13	1.381 (2)
C3—H3	0.9300	C12—H12	0.9300
C4—C5	1.373 (3)	C13—C14	1.369 (3)
C4—H4	0.9300	C13—Cl1	1.7459 (19)
C5—C6	1.393 (2)	C14—C15	1.366 (3)
C5—H5	0.9300	C14—H14	0.9300
C6—C7	1.498 (2)	C15—C16	1.385 (3)
C7—N1	1.4775 (19)	C15—H15	0.9300
C7—C8	1.532 (2)	C16—H16	0.9300
C7—H7	0.9800	C17—N1	1.461 (2)
C8—C18	1.470 (2)	C17—H17A	0.9600
C8—C9	1.537 (2)	C17—H17B	0.9600
C8—C10	1.567 (2)	C17—H17C	0.9600
C9—O1	1.423 (2)	C18—N2	1.129 (2)
C9—H9A	0.9700	N1—O2	1.4711 (17)
C9—H9B	0.9700		
			
O1—C1—C6	122.77 (14)	O2—C10—C11	109.49 (12)
O1—C1—C2	116.42 (15)	O2—C10—C8	104.48 (12)
C6—C1—C2	120.75 (17)	C11—C10—C8	115.63 (12)
C3—C2—C1	119.44 (19)	O2—C10—H10	109.0
C3—C2—H2	120.3	C11—C10—H10	109.0
C1—C2—H2	120.3	C8—C10—H10	109.0
C2—C3—C4	120.68 (19)	C12—C11—C16	119.17 (16)
C2—C3—H3	119.7	C12—C11—C10	121.27 (14)
C4—C3—H3	119.7	C16—C11—C10	119.56 (15)
C5—C4—C3	119.7 (2)	C13—C12—C11	119.03 (16)
C5—C4—H4	120.2	C13—C12—H12	120.5
C3—C4—H4	120.2	C11—C12—H12	120.5
C4—C5—C6	121.15 (19)	C14—C13—C12	122.17 (17)
C4—C5—H5	119.4	C14—C13—Cl1	118.99 (15)
C6—C5—H5	119.4	C12—C13—Cl1	118.84 (14)
C1—C6—C5	118.25 (16)	C15—C14—C13	118.59 (17)
C1—C6—C7	120.97 (14)	C15—C14—H14	120.7
C5—C6—C7	120.71 (15)	C13—C14—H14	120.7
N1—C7—C6	113.74 (12)	C14—C15—C16	120.76 (17)
N1—C7—C8	99.38 (11)	C14—C15—H15	119.6
C6—C7—C8	112.88 (12)	C16—C15—H15	119.6
N1—C7—H7	110.1	C15—C16—C11	120.27 (17)
C6—C7—H7	110.1	C15—C16—H16	119.9
C8—C7—H7	110.1	C11—C16—H16	119.9
C18—C8—C7	111.76 (12)	N1—C17—H17A	109.5
C18—C8—C9	109.29 (12)	N1—C17—H17B	109.5
C7—C8—C9	108.78 (12)	H17A—C17—H17B	109.5
C18—C8—C10	114.32 (13)	N1—C17—H17C	109.5
C7—C8—C10	102.39 (11)	H17A—C17—H17C	109.5
C9—C8—C10	110.04 (12)	H17B—C17—H17C	109.5
O1—C9—C8	112.07 (12)	N2—C18—C8	177.46 (19)
O1—C9—H9A	109.2	C17—N1—O2	104.46 (12)
C8—C9—H9A	109.2	C17—N1—C7	113.60 (13)
O1—C9—H9B	109.2	O2—N1—C7	100.14 (10)
C8—C9—H9B	109.2	C1—O1—C9	115.99 (12)
H9A—C9—H9B	107.9	C10—O2—N1	104.90 (11)
			
O1—C1—C2—C3	−177.20 (17)	C7—C8—C10—C11	−114.23 (14)
C6—C1—C2—C3	0.3 (3)	C9—C8—C10—C11	130.25 (14)
C1—C2—C3—C4	1.3 (3)	O2—C10—C11—C12	−28.6 (2)
C2—C3—C4—C5	−0.9 (3)	C8—C10—C11—C12	89.10 (18)
C3—C4—C5—C6	−1.0 (3)	O2—C10—C11—C16	152.35 (14)
O1—C1—C6—C5	175.18 (15)	C8—C10—C11—C16	−89.99 (18)
C2—C1—C6—C5	−2.2 (2)	C16—C11—C12—C13	0.7 (2)
O1—C1—C6—C7	−1.9 (2)	C10—C11—C12—C13	−178.38 (15)
C2—C1—C6—C7	−179.23 (15)	C11—C12—C13—C14	−0.4 (3)
C4—C5—C6—C1	2.5 (3)	C11—C12—C13—Cl1	−179.51 (13)
C4—C5—C6—C7	179.59 (17)	C12—C13—C14—C15	0.1 (3)
C1—C6—C7—N1	−98.81 (17)	Cl1—C13—C14—C15	179.19 (15)
C5—C6—C7—N1	84.21 (18)	C13—C14—C15—C16	−0.1 (3)
C1—C6—C7—C8	13.4 (2)	C14—C15—C16—C11	0.4 (3)
C5—C6—C7—C8	−163.53 (14)	C12—C11—C16—C15	−0.7 (3)
N1—C7—C8—C18	−158.11 (12)	C10—C11—C16—C15	178.42 (16)
C6—C7—C8—C18	81.06 (15)	C6—C7—N1—C17	−77.41 (16)
N1—C7—C8—C9	81.13 (13)	C8—C7—N1—C17	162.38 (12)
C6—C7—C8—C9	−39.70 (16)	C6—C7—N1—O2	171.80 (12)
N1—C7—C8—C10	−35.30 (13)	C8—C7—N1—O2	51.59 (12)
C6—C7—C8—C10	−156.14 (12)	C6—C1—O1—C9	20.4 (2)
C18—C8—C9—O1	−63.51 (17)	C2—C1—O1—C9	−162.18 (15)
C7—C8—C9—O1	58.76 (16)	C8—C9—O1—C1	−49.34 (18)
C10—C8—C9—O1	170.19 (12)	C11—C10—O2—N1	150.44 (12)
C18—C8—C10—O2	127.25 (13)	C8—C10—O2—N1	26.01 (14)
C7—C8—C10—O2	6.19 (14)	C17—N1—O2—C10	−167.72 (13)
C9—C8—C10—O2	−109.34 (13)	C7—N1—O2—C10	−49.94 (13)
C18—C8—C10—C11	6.83 (18)		
